# The relationship between circadian type and physical activity as predictors of cognitive performance during simulated nightshifts: A randomised controlled trial

**DOI:** 10.1080/07420528.2025.2503866

**Published:** 2025-05-19

**Authors:** Dayna Easton, Charlotte Gupta, Grace Vincent, Corneel Vandelanotte, Mitch Duncan, Philip Tucker, Lee Di Milia, Sally A. Ferguson

**Affiliations:** a Appleton Institute, Central Queensland University, Wayville, SA, Australia; b Active Living Research Program, Hunter Medical Research Institute, NSW, Australia; c School of Medicine & Public Health, The University of Newcastle, Callaghan, NSW, Australia; d School of Psychology, Swansea University, Swansea, UK; e Psychobiology and Epidemiology Division, Department of Psychology, Stockholm University, Stockholm, Sweden; f School of Business and Law, Central Queensland University, Rockhampton, QLD, Australia

**Keywords:** Shift work, night shift, cognitive performance, fatigue countermeasure, physical activity, individual difference, circadian type

## Abstract

Nightshift is associated with impaired cognitive performance on many tasks, yet performance is also moderated by individual differences. We investigated the effect of circadian type (two factors: flexible-rigid, and languid-vigour), and the efficacy of a novel countermeasure, breaking up sitting with light-intensity physical activity, in the context of nightshift performance. Thirty-three healthy adults (age M ± SD: 24.3 ± 4.6 y; 19 females) participated in a sleep laboratory study over five consecutive simulated nightshifts (2200–0600 h). Sleep opportunities occurred at 0800–1700 h. Participants were randomised to a sedentary (SIT; *n =* 14), or “breaking-up” sitting (BREAK; *n =* 19) condition. BREAK participants completed 3 min of light-intensity walking every 30 min at 3.2 km/h, while SIT participants remained seated. Every 2 h during nightshift, participants completed the Psychomotor Vigilance Task (mean RRT), Stroop Task, and Digit Symbol Substitution Task. Participants completed the revised Circadian Type Inventory which categorises individuals on a rigid-flexible scale and a languid-vigorous scale (rigid; *n =* 12, flexible; *n =* 11; languid; *n =* 11, vigorous *n =* 13). Linear mixed models showed a significant 3-way interaction between Nightshifts (1–5), Condition (SIT, BREAK), and flexibility-rigidity for mean RRT (*p =* 0.03) only. Flexible types in the BREAK condition had better performance than rigid BREAK, rigid SIT, and flexible SIT over five nights, with performance marginally worse on the first night for all participants apart from rigid SIT. Linear mixed models showed a significant 2-way interaction between Nightshifts (1–5), and flexibility-rigidity for percentage accuracy on the Stroop task, and a significant 2-way interaction between Nightshifts (1–5), and languid-vigour for response time on the Stroop task. Accuracy worsened for rigid types, while response time on the Stroop task improved for languid types over five nights. No other significant differences were found. Breaking up sitting with light-intensity physical activity maintained sustained attention for flexible circadian types across all five experimental nightshifts. Both rigidity and languidity moderated trends in performance, though whether these differences have meaningful real-world implications must be explored further. Our results indicate that circadian type classifications should be accounted for in breaking up sitting interventions overnight.

## Introduction

The current demands for 24-h services mean that non-standard work hours are the norm for a significant proportion of the Western workforce (Folkard and Tucker 2003; Shriane et al. 2020). Working during the night opposes intrinsic physiological sleep-wake cues and requires workers to maintain alertness and performance when sleep drive is high (Barger et al. 2009). Consecutive nightshifts exacerbate circadian misalignment – the mismatch between sleep and wake, the light dark cycle, and other circadian rhythms (i.e. behavioural, physiological, hormonal, cellular) (Chellappa et al. 2019; Ganesan et al. 2019; Haire et al. 2012; Huffmyer et al. 2016; James et al. 2021; Magee et al. 2016; McHill and Wright 2019) – which is associated with a wide array of adverse health and cognitive outcomes (Akerstedt and Wright 2009; Kecklund and Axelsson 2016). Working consecutive nightshifts is associated with impaired sustained attention on a psychomotor vigilance task (Waggoner et al. 2012), working memory on the Digit Symbol Substitution Task (Sunde et al. 2020), and response inhibition on the Stroop task (McHill and Wright 2019). There are potentially significant safety risks when performance on such cognitive domains is reduced during consecutive nightshifts (Brown et al. 2020; Chellappa et al. 2019; Johnson et al. 2014; Kecklund and Axelsson 2016). In practical settings, these risks can include preventable vehicle accidents in police officers (Barger et al. 2009; Vila et al. 2002) and bus drivers (Miyama et al. 2020), and needle-related injuries in nurses (Ayas et al. 2006). As such, mitigating the occupational risks that come with reduced cognitive capacity during consecutive nightshifts is essential.

Fatigue countermeasures designed to combat the cognitive impacts of consecutive nightshifts are well researched. These strategies include shift scheduling (e.g. forwards vs backwards rotation) (Garde et al. 2020; Niu et al. 2011), pharmacological treatments to improve sleep quality during the day (e.g. melatonin) (Burgess et al. 2002; Carriedo-Diez et al. 2022) and stimulants to increase alertness overnight (e.g. caffeine) (Centofanti et al. 2018, 2020). Other fatigue risk management strategies have involved frequent breaks lasting less than 10 min within a shift. Micro-break characteristics can include rest and relaxation, nutritional intake, socialisation, and cognitive activities (Kim et al. 2017; Tucker 2003). Frequent micro-breaks have been found to be beneficial at reducing fatigue (Albulescu et al. 2022), and improving attention (Mijović et al. 2015), task performance (Wendsche et al. 2016) and cognitive performance (Chandrasekaran et al. 2021) in day-workers. Further, incorporating light-intensity physical activity within these micro-breaks improved worker productivity and cognitive functions when compared to sedentary micro-breaks (Benatti and Ried-Larsen 2015; Chandrasekaran et al. 2021; Henning et al. 1997; Sallinen et al. 2008).

Recent literature has demonstrated that different modalities of physical activity to break up prolonged sitting at work differentially influence cognitive performance (Tuckwell et al. 2022). For instance, light-intensity walking interventions are reported to have greater efficacy in improving cognitive performance than sit-stand interventions, and may be more feasible than cycling desk interventions (Tuckwell et al. 2022). These studies have focused on day workers, and therefore, it is not known whether breaking up prolonged sitting with light-intensity walking is a suitable strategy for night workers. However, consecutive night work significantly reduces sleep quality and duration, worsening performance in shift workers (Chellappa et al. 2019; Kecklund and Axelsson 2016; Niu et al. 2011). In addition, night staff in some industries can be engaged in less physically demanding work than day staff (Chappel et al. 2017). In these instances, a physically active and alerting strategy, such as breaking up prolonged sitting, may be especially beneficial for cognitive performance and worker wellbeing (Vincent et al. 2017).

Examining whether breaking up prolonged sitting is effective at minimising the cognitive impacts of night work has important practical implications for occupational health and safety. However, the individual differences among shift workers suggest that not all countermeasures will be equally beneficial (Taillard et al. 2003; Van Dongen 2006). Variability in response to sleep restriction and tolerance to shift work has been found to be person-specific and related to trait-like characteristics in personality and circadian typology (Saksvik et al. 2011; Van Dongen 2006). Nightshift workers are often sleep restricted during their rotations (Johnson et al. 2014), and the person-specific nature of cognitive impairment in response to sleep loss will mean that performance differs between workers even under the same degree of sleep restriction (Van Dongen 2006). Circadian typology, or individual differences in circadian rhythm expression (Adan et al. 2012), can include subjective assessments of chronotype, which categorise people into morning, intermediate and evening types. Studies highlight that evening type shift workers accumulate less sleep debt the more consecutive nightshifts worked (Fischer et al. 2021) and have demonstrated greater improvements in subjective fatigue, sleepiness and mood in response to a field light-based intervention (Olson et al. 2020), compared to morning types. However, few studies have explored additional circadian typology measures in the context of objective performance and shift work.

Circadian type is an under-researched individual characteristic that could help to explain why some shift workers are better able to tolerate night work than others. Circadian type refers to individual differences in circadian rhythm function. Circadian rhythms can be assessed in terms of phase, amplitude and the stability of the amplitude (Baehr et al. 2000). While measures of phase have dominated the literature (Adan et al. 2012), the revised Circadian Type Inventory (rCTI; Di Milia et al. 2005) was developed to assess rhythm amplitude and stability. The rCTI consists of two factors. The first factor, rigid-flexible (FR), assesses rhythm stability and categorises individuals via the flexibility/rigidity of sleeping behaviours (Di Milia et al. 2005). Those who can adjust to irregular sleep-wake routines are classified as flexible types. The second factor, languid-vigour (LV), assesses rhythm amplitude via the languid/vigorous dimension, and refers to one’s ability to overcome drowsiness and fatigue, particularly following sleep restriction (Di Milia et al. 2005). Individuals who can overcome fatigue are considered vigorous. A systematic review examining the relationship between individual differences and shiftwork tolerance found that scoring high on flexibility and vigour was associated with greater subjective alertness on the night shift, while scoring high on languidity was not (Saksvik et al. 2011). Similarly, flexible types were less susceptible to alertness deficits during hours when sleep pressure typically increases, while vigorous types reported lower sleep need (Di Milia et al. 2005). These findings suggest that flexibility and vigour are associated with greater subjective tolerance to the challenges of nightshift (Saksvik et al. 2011). However, no current studies have examined the rCTI and objective performance measures in a shift working context. As such, it is unknown whether flexibility or vigour are associated with cognitive performance domains across consecutive nightshifts. It is also unknown whether circadian type influences the efficacy of fatigue countermeasures, such as physical activity (e.g. breaking up prolonged sitting with light-intensity walking), during nightshifts (Easton et al. 2024). Thus, the present study aims to examine the impact of circadian type on cognitive performance, and whether both breaking up sitting and circadian type impact the trends in cognitive performance over consecutive nightshifts.

## Research Questions

1. 1a. Does having either a flexible or rigid circadian type differentially impact performance on the Psychomotor Vigilance Task, the Stroop task and the Digit Symbol Substitution task over five nightshifts?
2. 1b. Does breaking up sitting differentially impact flexible and rigid circadian types, with respect to performance on the Psychomotor Vigilance Task, the Stroop task and the Digit Symbol Substitution task over five nightshifts?
3. 2a. Does having either a languid or vigorous circadian type differentially impact performance on the Psychomotor Vigilance Task, the Stroop task and the Digit Symbol Substitution task over five nightshifts?
4. 2b. Does breaking up sitting differentially impact languid or vigorous circadian types, with respect to performance on the Psychomotor Vigilance Task, the Stroop task and the Digit Symbol Substitution task over five nightshifts?

## Method

### Study Design

The present study was part of a broader in-laboratory, randomised controlled trial to determine the impact of simultaneous exposure to prolonged sitting, sleep restriction and circadian disruption, as well as the effects of breaking up sitting on these relationships (Vincent et al. 2020). The principal study included both day shift and nightshift conditions. Primary outcomes of the principal study included cognitive performance and cardiometabolic health outcomes. The principal study was conducted within the Appleton Institute Sleep Laboratory, CQUniversity Adelaide, was registered with the Australian New Zealand Clinical Trials Registry (12619001516178) and approved by the Central Queensland University Human Research Ethics Committee (0000021914). The complete protocol for the principal study is published separately (Vincent et al. 2020). The present study investigated whether breaking up sitting impacts cognitive performance over consecutive nightshifts differently according to circadian type.

### Participants

The principal study’s nightshift sample consisted of 52 healthy participants, 41 of whom completed the protocol (age M ± SD: 24.4 ± 4.6 y; 21 females; 23.4 ± 3.0 kg/m^2^). Participants aged between 18 and 35 y were recruited from Adelaide, South Australia via online advertisements, flyers and word of mouth. The circadian type of participants was determined using the 25th and 75th percentile scores on the flexible-rigid (FR) and languid-vigorous (LV) scales, and 33 participants (age 24.6 ± 4.8 y; 19 females; 23.3 ± 3.1 kg/m^2^) were included for analysis in the current study. The remaining participants did not fall into either flexible, rigid, languid or vigour classifications based on these cut-off scores and were not included in the analysis. Participants provided informed electronic consent to all the study requirements, such as limited physical activity and phone use, and abstinence from caffeine and alcohol during the laboratory protocol. Participants received compensation following study completion (AUD$780). Eligibility was determined via a battery of standardised and validated questionnaires. Inclusion criteria included body mass index (BMI) between 18 and 30 kg/m^2^, low-habitual users of caffeine and alcohol, non-smokers, low physical activity levels (sitting ≥5 h/d and having ≤150 min/week of moderate-intensity exercise for >3 months, as screened by the International Physical Activity Questionnaire), no history of contraindications to physical activity, no transmeridian travel within the past 3 months, no current shift work, no diagnosed psychiatric or sleep disorders, and habitual bed (22:00–00:00 h) and wake times (06:00–08:00 h) (Vincent et al. 2020). Female participants provided menstrual cycle information during this screening process. Based on these data, cycle timing was calculated to ensure participants’ luteal phase aligned with study periods. The full eligibility criteria were also reported within the study protocol (Vincent et al. 2020). Participant demographics by condition are reported below (see Table 1).

**Table 1.** Participant demographics according to circadian type categorisation (flexible, rigid, languid, vigorous). Data are presented as mean ± standard deviation.

Condition	N	Sex	Age (y)	BMI (kg/m^2^)	Height (m)
Flexible	4	Female	23.8 ± 4.3	20.7 ± 1.6	1.64 ± 0.08
	7	Male	22.3 ± 4.2	24.4 ± 1.6	1.82 ± 0.08
Rigid	6	Female	23.8 ± 4.0	21.1 ± 3.1	1.64 ± 0.08
	6	Male	25.2 ± 6.3	23.7 ± 3.6	1.78 ± 0.06
Languid	6	Female	25.0 ± 3.7	23.0 ± 4.2	1.61 ± 0.10
	5	Male	22.0 ± 4.7	25.6 ± 2.8	1.73 ± 0.10
Vigorous	7	Female	26.1 ± 5.2	22.8 ± 3.2	1.65 ± 0.06
	6	Male	26.0 ± 5.8	23.2 ± 3.0	1.85 ± 0.09
Total	33		24.2 ± 4.7	23.4 ± 3.2	1.71 ± 0.12

Note. Participants who are categorised as more than one circadian type are only included once in the reported total.

### Study Conditions

**Figure 1.**
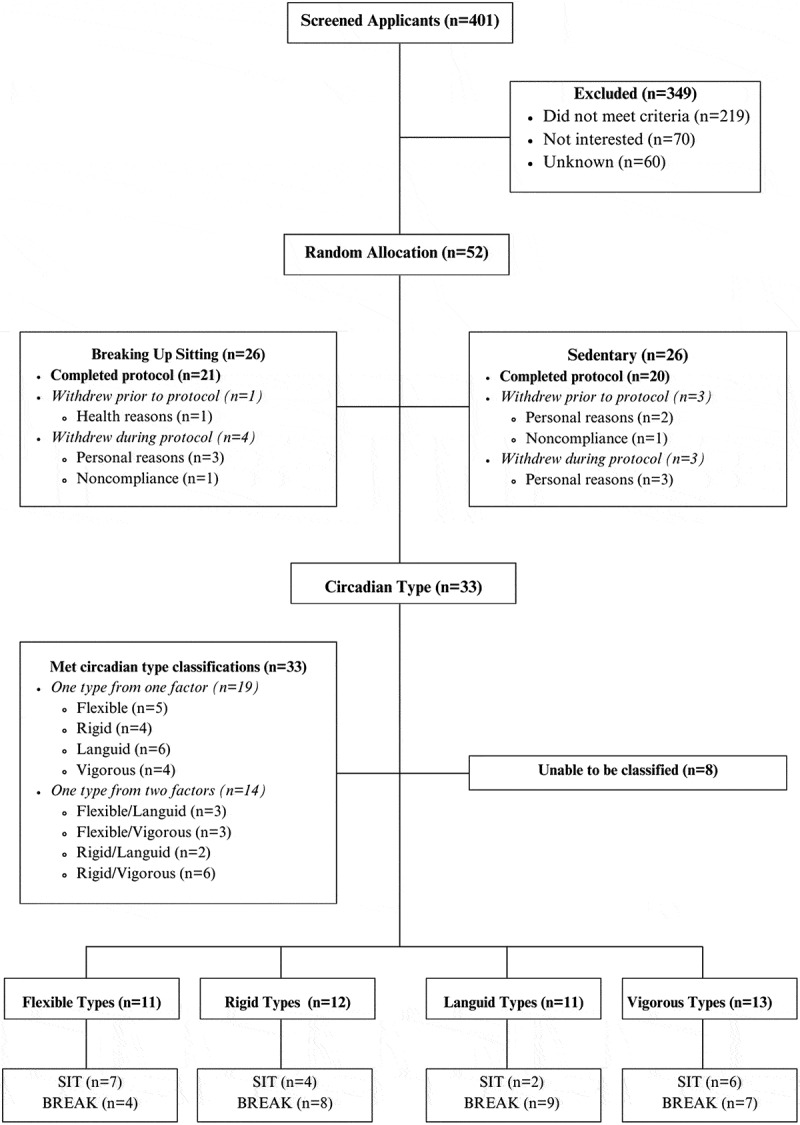
Consort diagram. SIT = sedentary; BREAK = breaking up sitting.

Participants (*n* = 41) were randomly allocated into either the sedentary (SIT) (*n* = 20) or breaking up sitting condition (BREAK) (*n* = 21) prior to study admission. A block randomisation strategy was conducted with a block size of 6 (the number of individual bedroom suites in the Appleton Sleep Laboratory) to ensure participants were randomly allocated to either physical activity condition: Breaking up Sitting (3 participants) or Sedentary (3 participants). After the first six participants in the study, all subsequent participants were stratified by sex (to ensure equal number of males and females in the physical activity conditions) and BMI (to ensure no statistical difference in mean BMI of each physical activity condition). Randomisation was conducted by the research team, and participants were not blinded to study condition. Participants completed the two-factor 11-item revised Circadian Type Inventory (rCTI; factor 1: flexible or rigid; factor 2: languid or vigorous). Participants could either fall into one subgroup of one factor (e.g. flexible only) or into one subgroup of each factor (e.g. flexible and vigorous). However, participants could not be in two subgroups from the same factor (e.g. flexible and rigid). The four subgroups were determined using the 25th and 75th percentile scores on the FR and LV scales (Di Milia et al. 2004, 2005); flexible (75th percentile; *n* = 11), rigid (25th percentile; *n* = 12), languid (75th percentile; *n* = 11), and vigorous (25th percentile; *n* = 13) (See Figure 1). The limited number of participants in the SIT languid subgroup (SIT = 2, BREAK = 9) meant that it was not possible to examine the interaction between condition (SIT vs BREAK) and LV (Research Question 2a).

### Laboratory Setting

Participants lived at the Appleton Institute Sleep Laboratory in Adelaide (Australia) for the 7-d protocol. The facility has six sound-attenuated and temperature-controlled bedrooms with a king-single bed and personal ensuite, and two living/kitchen areas. In line with previous research recommendations, temperature and humidity were maintained at 21°C + 2°C and 40–60%, respectively (Cao et al. 2021; Harding et al. 2019). The lighting system was consistent with typical office lighting and maintained a constant broad-spectrum white light of >300 lux without manual adjustments during wake periods. Illuminance was measured via the CENTER 530 Light Meter (CenterTek, Taiwan) positioned horizontally (sensor facing upward) at seated eye level, consistent with participants’ posture during wake periods. Lux levels were negligible (<0.3 lux) during sleep periods. Light exposure was not measured throughout the experimental week as the system ensured that lighting conditions remained constant.

### Protocol

**Figure 2.**
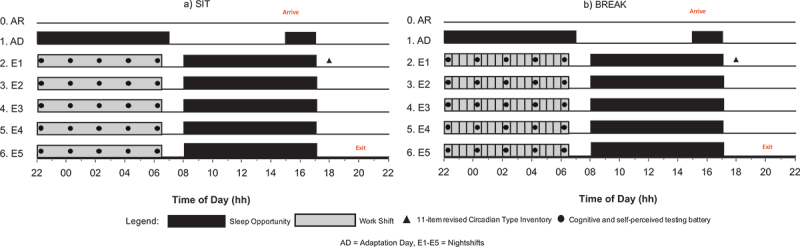
Protocol diagram displaying the sleep periods, activity periods across time (x-axis) and nightshifts (y-axis). Panel a shows the protocol for sitting (SIT), and panel b shows the protocol for breaking up sitting (BREAK). AR, arrival Evening; AD, adaptation Day; E1 to E5, nightshifts.

This protocol consisted of one Arrival Evening (AR), one Adaptation Day (AD) and five Nightshifts (E1-E5). The sleep opportunity following the completion of E5 served as a recovery sleep (see Figure 2). Participants were required to maintain a consistent bedtime between 22:00 and 00:00 h and wake time between (06:00–08:00 h) for 1 week prior to entering the laboratory. Adherence was monitored through daily check-ins and was confirmed by sleep diaries, actigraphy and physical activity monitoring (ActivPal). Participants were asked to abstain from caffeine, alcohol and strenuous physical activity 48 h (confirmed via ActivPal) prior to the study commencement. The full pre-experimental protocol for all participants is reported elsewhere (Vincent et al. 2020).

Participants arrived at 17:00 on the Arrival Evening, after which a briefing on protocol and procedures occurred. Participants slept from 22:00 to 07:00 on the Arrival Evening to facilitate adjustment to the laboratory environment. The following nightshifts involved 9-h sleep opportunities, starting at 08:00 and ending at 17:00. On Adaptation Day, participants underwent familiarisation with the cognitive and self-perceived capacity battery. The rCTI was administered at 18:00 on Adaptation Day as part of a comprehensive one-off questionnaire battery. A nap opportunity from 15:00 to 17:00 was provided prior to commencing the first nightshift (E1).

From E1 to E5, participants performed simulated 8-h nightshifts from 22:00 until 06:00. The use of mobile phones was limited to 1.5 h each evening at 7:00 pm, 3 h prior to the start of each simulated night shift. This use occurred under bright light conditions. Standard iso-caloric meals based on height and weight were provided during each nightshift based on a Western diet and included a dinner meal prior to the shift, a lunch meal during the shift, and a breakfast meal following the shift. This meal timing was designed to reflect the real shift worker dietary habits (Gupta et al. 2019). Participants pre-selected lunch and dinner meals from a predetermined list prior to study commencement, while snacks and breakfast remained consistent. All participants engaged in sedentary activities such as watching TV and reading throughout the night. During the simulated shift, participants allocated to BREAK were required to undergo 3 min of light-intensity physical activity every 30 min. The physical activity involved walking at a light pace of 3.2 km/h on a motorised treadmill (Healthrider H95T; Icon Health & Fitness Inc, Utah, USA) at a 0% gradient. All treadmill parameters aligned with the current literature regarding light-intensity exercise as a strategy to improve health and cognitive outcomes (Bailey and Locke 2015). The breaks occurred from the beginning of the nightshift (22:00), with the final walk coinciding with the end of the shift (06:00). In total, participants in the BREAK condition performed 17 bouts of breaking up sitting each night shift. Outside of designated intervention sessions, participants in the BREAK condition remained sedentary. Participants allocated to the SIT condition remained sedentary throughout the study. On each nightshift (E1-E5), the cognitive performance and self-perceived capacity of participants were measured via five testing batteries, every 2 h (22:00, 00:00, 02:00, 04:00, 06:00), following the designated walk at that time.

## Measures

### Control Variables

#### Physical Activity Monitoring

Triaxial accelerometers were worn to ensure compliance with intervention requirements (i.e. sedentary or breaking up sitting). The activPal monitors (PAL Technologies, Glasgow, Scotland) are approximately 24 × 43 × 5 mm in size and weigh 9 g. The devices were encased in a nitrile sleeve and positioned on the anterior midline of the right thigh by a waterproof adhesive dressing. Activity duration and type, step count, sit-to-stand ratios, and time spent sitting and lying are all measurable via this monitor. Step count alone was included in the final analysis to demonstrate group differences in activity levels. In line with the existing literature, data was measured at 40 hz (Stanton et al. 2014).

#### Objective Sleep

Objective sleep was assessed using polysomnography (PSG) and standard electroencephalography (EEG) via the Compumedics Grael PSG/EEG system (Compumedics Grael; Melbourne, Victoria, Australia). A derivation of electrodes (i.e. F4-M1, C3-M2 and O2-M1) was used concurrently with two electrooculograms (placed on the left and right outer canthi) and three electromyograms (one placed in the middle of the mandible, and two placed 2 cm below the inferior edge of the mandible). Sleep records were blinded, recorded in 30-s epochs, and scored by the same technician according to AASM scoring criteria (Iber et al. 2007). The following variables were calculated following each sleep period: (i) total sleep time (TST), (ii) wake after sleep onset (WAS), (iii) sleep efficiency, (iv) sleep onset latency (SOL), (v to viii) and the time spent in Stages N1, N2, N3 and rapid eye movement (REM).

### Main Variables

#### Circadian Type

Circadian type was assessed using the 11-item Revised Circadian Type Inventory (rCTI). The CTI assesses the degree to which people can alter their sleeping behaviours and daily preferences (Di Milia et al. 2005). The rCTI has good psychometric properties. Corrected item – total correlation for the five item FR scale ranged from 0.51 to 0.68, and from 0.36 to 0.50 for the LV scale. Cronbach's alpha for the FR scale was 0.80, and 0.68 for the LV scale. Test–retest reliability (*n* = 178) after 3 months was 0.75 for the FR scale and 0.72 for the LV scale. Using structural equation modelling, the factor structure was confirmed in a working sample (Di Milia et al. 2005), and in a random population sample (Di Milia and Folkard 2021). A sample item from the FR scale is: “Do you find it as easy to work late at night as earlier in the day?” and from the LV scale: “Do you find it difficult to “‘wake-up’” properly if you are awoken at an unusual time?” (Di Milia et al. 2005). Each item is rated on a 5-point Likert scale; 1 = almost never, 2 = seldom, 3 = sometimes, 4 = usually, 5 = almost always.

#### Sustained Attention

Sustained attention was assessed using a 10-min psychomotor vigilance task (PVT) to quantify participants’ reaction time (PVT-192, Ambulatory Monitoring Inc., Ardsley, New York). The PVT is highly sensitive to circadian misalignment and is a well-validated measure of fatigue-related cognitive performance (Chellappa et al. 2019; Dorrian et al. 2004; Killgore 2010; Van Dongen et al. 2003). This task has high validity and is highly reliable with test–retest reliability intra-class correlations for metrics such as lapses and median response time measuring above 0.8 (Dorrian et al. 2004). The task was performed on a hand-held device featuring an LED display and two response buttons. Participants were tasked to respond immediately to presentations of a stimulus at varied 2–10 s intervals. Participants were required to complete three 10-min PVTs on Adaptation Day in accordance with the literature on practice effects (Killgore 2010). The outcome variables extracted from the PVT were reciprocal reaction time (RRT), and lapses of more than 500 ms. Mean reciprocal reaction time is calculated as 1/response time, e.g. 1/250s. Higher scores for mean RRT indicate better performance.

#### Response Inhibition

Response inhibition was assessed via the Stroop task. This task quantifies the ability to inhibit one’s responses in the presence of cognitive interference (Stroop 1935, 1992). The literature has demonstrated that the task is highly sensitive to changes in sleep behaviour (Stenuit and Kerkhofs 2008). It requires participants to respond to the colour of a word presented on a screen by pressing a corresponding coloured button on a keypad. The present study used a reversal of the standard Stroop protocol. The usual task requires participants to respond to the font colour, while our task required participants to respond to the word itself. The word may be presented in a font colour that does not correspond to the written word. For instance, “RED” may be presented in green font, whereby participants would respond with “RED.” This written word could also be presented in the corresponding font colour in which case the correct response again is “RED.” Doing this removes the possibility for participants to use a strategy where peripheral vision is used to focus on the colour, not the written text (Cain et al. 2011). Derivatives of this task used for analysis were percentage correct and mean reaction time (ms).

#### Working Memory

The Digit Symbol Substitution Task (DSST) was used to assess working memory. The task also assesses response speed and visuomotor coordination (Jaeger 2018). The task consists of nine digits (0 to 9) which correspond to unique predetermined symbols which were randomly ordered. Participants were required to correctly draw the associated symbols for each digit and were not permitted to skip any digits. Participants were given 90 s to complete as many symbols as possible. Participants underwent 14 familiarisation trials to avoid learning effects. The outcome variable of the DSST was the number of correct responses (accurate and legible) completed in the 90 s. All DSSTs were scored by the same researcher and then double-entered.

## Statistical Analyses

Data were analysed via Jamovi 2.3.18.0 (2023). For the LV factor, the limited number of participants in the SIT languid subgroup (SIT; *n* = 2, BREAK; *n* = 9) meant that sedentary participants could not be compared between groups for any control or outcome analyses. Thus, only participants in languid and vigour BREAK were included in the final analyses of the LV factor. Residuals were checked for normality. All data are reported as Mean ± Standard error of the mean unless specified and the statistical significance set at *p* = 0.05.

### Control Variables (Sex, BMI, Steps, Sleep)

Analyses were conducted to verify that randomisation stratified by sex and BMI had achieved equal distributions of these variables between the two conditions. We compared the numbers of men and women in the two conditions by conducting separate independent *t*-tests (with sex as a binary outcome) for the FR comparison and for the LV comparison. No significant differences were found for either comparison, and so sex was not included as a covariate in the outcome models. Likewise, to compare BMI values in the two conditions, two separate independent *t*-test were conducted for the FR comparison and for the LV comparison. No significant differences were found for either comparison and so BMI was not included as a covariate in the outcome models.

An independent *t*-test was conducted for total daily step count to confirm a statistical difference (by design) between the participants in SIT and BREAK conditions. Total daily step count was assessed within circadian type BREAK, and circadian type SIT conditions to ensure that physical exertion was not significantly different and confounding performance data. A linear mixed model was fitted for the FR comparison, with circadian type (F, R), condition (SIT, BREAK), and their interactions were included as fixed factors. No significant effects were observed and so step count was not included as a covariate in outcome models. For the LV comparison, a *t*-test was conducted to ensure that the total daily step count did not differ between conditions. A significant difference was observed and so total daily step count for the LV comparison was included as a covariate in all performance outcome models.

To examine differences in polysomnographic sleep data for the FR comparison, a series of separate linear mixed models were fitted for each sleep variable. Circadian type (F, R), condition (SIT, BREAK) and their interactions were included as fixed factors. No significant effects were observed for the FR comparison and so sleep variables were not included as covariates. For the LV comparison, a *t*-test revealed significant differences between conditions for TST, WASO, SE and Stage N2. Based on these significant differences, TST, WASO, SE and Stage N2 were also included as covariates in the LV BREAK outcome analyses.

### Main Variables

Separate mixed models were run for the two factors, FR and LV. For each factor, separate linear mixed models were fitted for the Psychomotor Vigilance task, the Stroop task, and the Digit Symbol Substitution task. For the FR comparison, the analyses examined the effects of circadian type and breaking up sitting on performance. For the LV, the analyses examined the effect of circadian type only.

### Flexible-Rigid Comparison

For the FR factor, separate linear mixed models were fitted on all performance outcome variables. Circadian type (F, R), condition (SIT, BREAK) and nightshifts (E1-E5) and their interactions were included as fixed factors. These fixed effects were the same for all cognitive performance variables. Participant ID was included as a random effect to account for repeated measures on individuals.

### Languid-Vigorous Comparison

For the LV factor, separate linear mixed models were fitted on all performance outcome variables. Circadian type (L, V), nightshifts (E1-E5), and their interactions were included as fixed factors. These fixed effects were the same for all cognitive performance variables. Participant ID was included as a random effect to account for repeated measures on individuals. Step count, TST, WASO, SE and Stage N2 were included as covariates in all performance outcome analyses.

## Results

### Physical Activity Monitoring

No significant two-way interaction between circadian type × condition for step count was observed, *F*(1,18.0) = 2.79, *p =* 0.112. Daily average step count was different (by design) between SIT (1078 ± 46.94 steps) and BREAK (6464 ± 60.23 steps), *t*(144) = 64.6, *p =* < .001. A significant difference was observed between BREAK languid (6724 ± 84.5) and BREAK vigorous types (6151 ± 77.1), *t*(77) = 4.85, *p =* < .001.

To further understand why these differences existed, height differences (cm) were explored. Languid participants were significantly shorter (166 ± 1.71 cm) than vigorous participants (178 ± 1.83 cm), *t*(78.0) = −4.87, *p =* < .001. Step length was then calculated as a function of height to determine if this was contributing to differences in total daily step count between languid and vigorous participants. Step length differed significantly between languid (56.17 ± 0.71 cm) and vigorous types (59.87 ± 0.96 cm), *t*(76.0) = 3.14, *p =* .002.

### Objective Sleep

Sleep variables of interest were not significantly different between flexible and rigid types in either SIT or BREAK (see Table 2). For languid and vigorous types, significant differences in TST (*p* = <.001), WASO (*p* = <.001), SE (*p* = <.001), N2 (*p* = 0.011) were observed. All other sleep variables of interest did not significantly differ between languid and vigorous types (see Table 2).

**Table 2.** All sleep variables for: the two-way interaction between physical activity condition (SIT, BREAK) by circadian type (F, R); and the mean difference between languid and vigour. Statistical significance set at *p* = 0.05.

Sleep Variable	Flexible		Rigid		f	df	p	Languid	Vigour	t	df	p
	Sit	Break	Sit	Break				Break	Break			
TST (min)	481.00 ± 56.68	468.00 ± 24.80	440.00 ± 18.4	428.00 ± 17.70	5.854	1, 18.6	0.981	464.00 ± 7.36	413.00 ± 9.92	4.040	68.0	<.001
WASO (min)	47.30 ± 15.6	65.70 ± 24.70	91.0 ± 18.10	106.4 ± 17.5	0.005	1, 18.8	0.939	69.3 ± 6.60	122.00 ± 10.0	−4.317	68.0	<.001
SE (%)	90.10 ± 3.00	86.70 ± 4.73	81.50 ± 3.49	79.20 ± 9.08	0.023	1, 18.8	0.880	85.90 ± 1.36	76.50 ± 1.84	4.042	68.0	<.001
SOL (min)	5.79 ± 1.29	6.07±.95	5.43 ± 1.50	5.60 ± 1.40	0.001	1, 17.9	0.972	7.06 ± 1.47	4.94 ± 0.71	1.319	68.0	0.192
N1 (min)	28.50 ± 5.20	30.33 ± 7.21	22.20 ± 5.20	21.70 ± 5.11	0.040	1, 18.8	0.843	22.80 ± 1.67	24.30 ± 1.60	−0.630	68.0	0.531
N2 (min)	223.00 ± 13.4	203.00 ± 21.20	211.00 ± 15.6	172.00 ± 15.1	0.339	1, 18.1	0.685	197.00 ± 7.76	166.00 ± 8.58	2.612	68.0	0.011
N3 (min)	119.00 ± 10.90	124.00 ± 17.5	109.00 ± 12.70	129.00 ± 12.40	0.341	1, 18.4	0.566	142.00 ± 6.58	130.00 ± 4.62	1.610	68.0	0.112
REM (min)	109.90 ± 19.30	111.00 ± 13.22	98.00 ± 9.79	109.90 ± 9.39	0.080	1, 18.3	0.780	102.00 ± 4.45	93.20 ± 4.48	1.374	68.0	0.174

*Note*. TST, total sleep time; WASO, wake after sleep onset; SE, sleep efficiency; SOL, sleep onset latency; N1, stage 1; N2, stage 2; N3, stage 3; REM, rapid eye movement. Data are presented as mean ± SE in minutes (with the exception of sleep efficiency presented as a percentage).

### Main Variables: Flexible-Rigid

#### Sustained Attention: Psychomotor Vigilance Task

##### Mean Reciprocal Reaction Time (RRT)

The two-way interaction between circadian type and nightshifts was not significant (*p =* .145; See Tables 3 and 4).

**Table 3.** Results from the mixed effects analysis of variance (ANOVA) showing the main effects of physical activity condition (SIT, BREAK), circadian type (FR), and nightshifts (E1-E5), and the interaction effects of physical activity condition by circadian type, physical activity condition by nightshifts, circadian type by nightshifts, and physical activity condition by circadian type by nightshifts.

	Physical Activity Condition (SIT, BREAK)		Circadian Type		Nightshifts		Physical Activity Condition*Circadian Type		Physical Activity Condition* Nightshifts		Circadian Type* Nightshifts		Physical Activity Condition*Circadian Type* Nightshifts	
Flexible-Rigid	F_(df)_	*p*	F_(df)_	*p*	F_(df)_	*p*	F_(df)_	*p*	F_(df)_	*p*	F_(df)_	*p*	F_(df)_	*p*
**PVT**														
Mean RRT	9.42_(1, 19.00)_	**0.006**	11.64_(1, 19.00)_	**0.003**	3.47_(4, 447.00)_	**0.008**	0.92_(1, 19.00)_	0.349	0.63_(4, 447.00)_	0.638	1.72_(4, 447.00)_	0.145	2.56_(4, 447.00)_	**0.038**
Lapses	3.97_(1, 21.80)_	0.059	4.31_(1, 19.30)_	0.052	1.03_(4, 447.40)_	0.390	0.39_(1, 21.80)_	0.534	0.87_(4, 447.40)_	0.482	0.78_(4, 447.40)_	0.537	1.02_(4, 447.40)_	0.398
**DSST**														
Correct Responses	0.49_(1, 19.00)_	0.489	8.18_(1, 19.00)_	**0.010**	5.11_(4, 456.00)_	**<0.001**	0.19_(1, 19.00)_	0.666	1.34_(4, 456.00)_	0.253	1.08_(4, 456.00)_	0.363	1.73_(4, 456.00)_	0.143
**Stroop**														
Percentage Correct	0.15_(1, 19.00)_	0.701	0.43_(1, 19.00)_	0.516	7.53_(4, 452.00)_	**<0.001**	0.08_(1, 19.00)_	0.778	2.27 _(4, 452.00)_	0.060	2.65_(4, 452.00)_	**0.032**	1.01_(4, 452.00)_	0.400
Mean Response Time	0.20_(1, 19.00)_	0.658	7.29_(1, 19.00)_	**0.014**	25.10_(4, 452.00)_	**<0.001**	1.18_(1, 19.00)_	0.290	0.63_(4, 452.00)_	0.639	1.09_(4, 452.00)_	0.358	0.83_(4, 452.00)_	0.504

*Note*. PVT = Psychomotor Vigilance Task; RRT = reciprocal reaction time; DSST = Digit Symbol Substitution Task. Numbers in bold indicate a level of statistical difference: p ≤ 0.05.

**Table 4.** Means and 95% confidence intervals for all cognitive performance outcomes for flexible, rigid, flexible SIT, rigid SIT, flexible BREAK and Rigid BREAK for each nightshifts.

Nightshifts						
	Flexible	Rigid	Flexible SIT	Rigid SIT	Flexible BREAK	Rigid BREAK
**PVT (Mean RRT)**						
E1	4.29(3.95–4.63)	3.66(3.34–3.97)	3.82(3.41–4.23)	3.48(3.04–3.93)	**4.76(4.21–5.30)**	3.83(3.39–4.28)
E2	4.45(4.11–4.79)	3.68(3.36–3.99)	4.03(3.62–4.44)	3.48(3.04–3.93)	**4.87(4.33–5.41)**	3.87(3.42–4.31)
E3	4.37(4.03–4.71)	3.57(3.26–3.89)	3.90(3.49–4.31)	3.35(2.90–3.79)	**4.83(4.29–5.37)**	3.80(3.35–4.24)
E4	4.33(3.99–4.67)	3.58(3.26–3.89)	**3.94(3.53–4.36)**	**3.27(2.82–3.71)**	**4.72(4.18–5.27)**	3.89(3.45–4.34)
E5	4.42(4.08–4.76)	3.65(3.33–3.96)	3.96(3.53–4.37)	3.40(2.96–3.85)	**4.88(4.33–5.42)**	3.89(3.45–3.34)
**PVT (Lapse count)**						
E1	1.56(−0.78–3.91)	3.83(1.67–5.99)	2.82(5.25–5.66)	4.80(1.74–7.85)	0.30(−3.44–4.04)	2.86(−0.18–5.92)
E2	1.18(−1.16–3.53)	4.17(2.00–6.33)	1.91(−0.91–4.74)	5.03(1.97–8.09)	0.45(−3.29–4.19)	3.30(0.24–6.35)
E3	1.91(−0.43–4.25)	4.43(2.27–6.59)	2.71(−0.11–5.54)	5.33(2.27–8.39)	1.10(−2.64–4.84)	3.53(0.47–6.59)
E4	1.84(−0.58–4.18)	4.76(2.57–6.95)	2.57(−0.25–5.40)	6.72(3.58–9.87)	1.10(−2.64–4.84)	2.80(−0.25–5.85)
E5	1.76(−0.58–4.11)	3.29(1.10–5.46)	2.57(−0.25–5.40)	3.62(0.55–6.69)	0.95(−2.79–4.69)	2.95(−0.14–6.05)
**Stroop (Percentage correct)**						
E1	98.01(97.1–98.8)	**98.23(97.2–99.1)**	97.55(96.3–98.8)	98.02(96.9–99.2)	98.43(97.2–99.7)	98.36(96.8–99.8)
E2	97.93(97.0–98.8)	**97.93(96.9–98.8)**	97.68(96.3–98.8)	97.81(96.7–99.0)	98.36(97.0–99.5)	98.04(96.4–99.5)
E3	98.03(97.1–98.9)	**97.26(96.2–98.1)**	97.62(96.3–98.8)	97.25(96.1–98.4)	98.48(97.1–99.6)	97.13(95.6–98.6)
E4	98.17(97.2–99.0)	**97.54(96.5–98.4)**	98.11(96.8–99.3)	97.37(96.1–98.4)	98.12(96.9–99.4)	97.77(96.2–99.2)
E5	97.54(96.6–98.3)	**96.81(95.8–97.7)**	97.76(96.5–98.9)	97.09(95.8–98.1)	97.24(96.0–98.5)	96.85(95.0–98.1)
**Stroop (Mean response time)**						
E1	0.77(0.68–0.87)	0.91(0.82–0.99)	0.79(0.68–0.91)	0.88(0.75–1.00)	0.75(0.60–0.90)	0.93(0.81–1.06)
E2	0.72(0.62–0.81)	0.88(0.79–0.99)	0.73(0.62–0.85)	0.85(0.72–0.97)	0.71(0.55–0.86)	0.92(0.79–1.04)
E3	0.69(0.60–0.79)	0.87(0.78–0.96)	0.71(0.60–0.83)	0.81(0.69–0.94)	0.67(0.52–0.82)	0.92(0.80–1.05)
E4	0.67(0.58–0.77)	0.85(0.76–0.94)	0.69(0.58–0.81)	0.79(0.67–0.91)	0.66(0.50–0.81)	0.91(0.79–1.03)
E5	0.65(0.55–0.74)	0.81(0.73–0.90)	0.67(0.55–0.78)	0.76(0.64–0.89)	0.63(0.47–0.78)	0.87(0.74–0.99)
**DSST (Number of correct responses)**						
E1	50.82(39.8–61.9)	36.47(26.3–46.6)	50.31(37.0–63.7)	39.02(24.6–53.3)	51.34(33.7–68.9)	33.91(19.5–48.2)
E2	58.46(47.4–69.5)	36.92(26.7–47.0)	60.63(47.3–73.9)	37.07(22.7–51.4)	56.27(38.6–73.8)	36.72(22.3–51.1)
E3	57.39(46.3–68.4)	39.74(29.5–49.8)	64.05(50.7–77.3)	44.11(29.7–58.4)	50.72(33.1–68.3)	35.24(20.9–49.6)
E4	59.37(48.3–70.4)	39.42(29.2–49.5)	63.97(50.5–77.2)	38.49(24.0–52.8)	54.86(37.2–72.4)	40.33(26.0–54.7)
E5	64.91(53.9–76.0)	42.38(32.1–52.4)	71.33(58.0–84.6)	40.62(26.2–54.9)	58.66(41.0–76.2)	43.91(29.6–58.3)

*Note*. PVT = Psychomotor Vigilance Task; RRT = reciprocal reaction time; DSST = Digit Symbol Substitution Task; E1-E5 = Nightshifts 1–5. Numbers in bold indicate a level of statistical difference: *p* ≤ 0.05.

**Figure 3.**
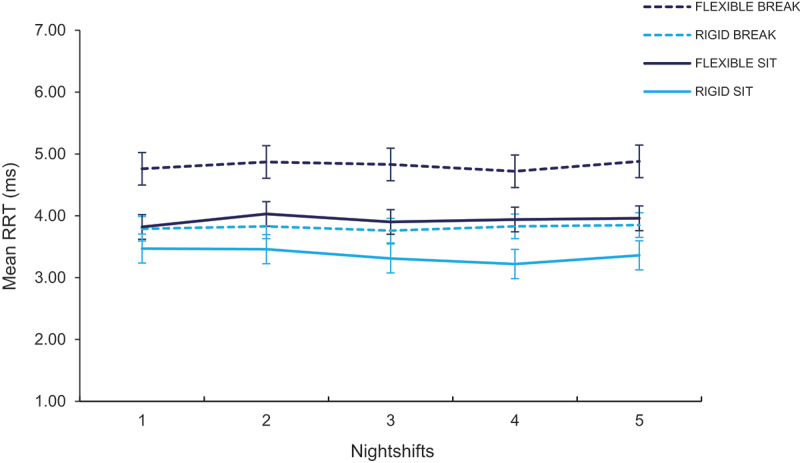
Mean reciprocal reaction time (ms) on the psychomotor vigilance task across five consecutive nightshifts for flexible and rigid types, by activity condition (SIT, BREAK). Higher scores indicate faster performance. Error bars presented as standard error.

The three-way interaction between condition × nightshifts × circadian type was significant (see Tables 3 and 4). Across all Nightshifts, participants in flexible BREAK had faster mean RRT than participants in rigid BREAK, flexible SIT and rigid SIT, with all participants except Rigid SIT performing worse on the first night (see Figure 3, see Table 4). There were significant differences between participants in flexible SIT and rigid SIT on E4 only (*p* = 0.030). Mean RRT was not significantly different for participants in Rigid SIT and Rigid BREAK. The main effect of condition on mean RRT was significant (*p =* .006) such that the participants in SIT (3.66 ± 0.14) had significantly slower response time than BREAK (4.33 ± 0.16; See Table 3). The main effect of nightshifts was significant (*p = *.008) such that the performance was better on E2 (4.06 ± 0.11) compared to E4 (3.96 ± 0.11; *p = *.030) but not E1, E3 or E5. The main effect of circadian type was significant (*p* = 0.003) such that the response time was faster for flexible types (4.37 ± 0.16) than rigid types (3.63 ± 0.15).

##### Lapse Count

The two-way interaction between circadian type × nightshifts on lapse count was not significant (*p =* .537; See Tables 3 and 4). The three-way interaction between condition × nightshifts × circadian type was not significant (*p =* .398; See Table 3). The main effects of circadian type (*p =* .052), nightshifts (*p =* .390) or condition (*p =* .059) were not significant.

#### Response Inhibition: Stroop

##### Percentage Correct

**Figure 4.**
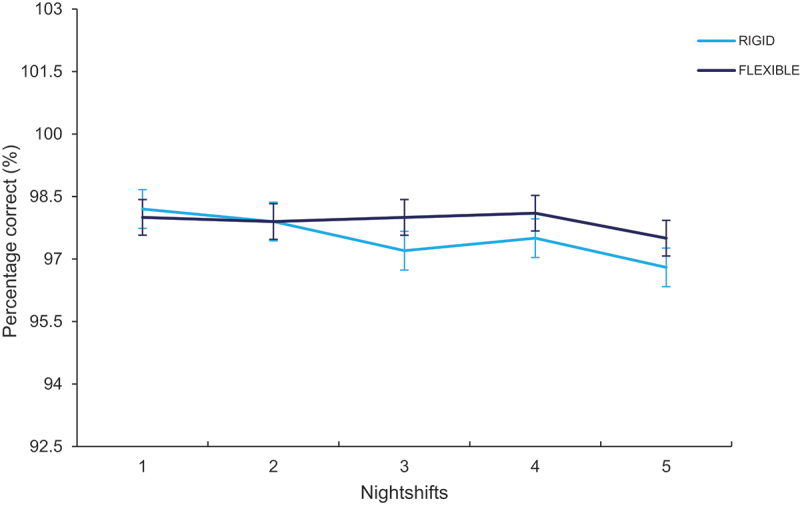
Percentage correct (%) responses on the Stroop task across five consecutive nights for flexible and rigid types. Error bars presented as standard error.

The two-way interaction between circadian type × nightshifts was significant, such that the percentage accuracy decreased in rigid types after five nights (*p =* .032; See Figure 4). Conversely, there was no significant change in flexible types. The three-way interaction between condition × nightshifts × circadian type was not significant (*p =* .798; See Table 3; See Table 4). The main effects of circadian type (*p =* .516) and condition (*p =* .701) were not significant. The main effects of nightshifts were significant (*p =* < .001), such that the correct responses decreased across nightshifts from E1 (98.13 ± 0.31) to E5 (97.10 ± 0.31).

##### Mean Response Time

The two-way interaction between circadian type × nightshifts on mean response time was not significant (*p =* .145; See Tables 3 and 4). The three-way interaction between condition × nightshifts × circadian type was also not significant (*p =* .834; See Table 3). The main effects of circadian type (*p =* .014) and nightshifts (*p =* < .001) were significant, such that the response time was faster in flexible types (0.70 ± 0.04) than rigid types (0.87 ± 0.04) and worsened across experimental nights from E1 (0.83 ± 0.03) to E5 (0.73 ± 0.03). The main effects of condition were not significant (*p =* .377).

#### Working Memory: Digit Symbol Substitution Task

##### Number of Correct Responses

The two-way interaction between circadian type × nightshifts was not significant (*p =* .358; See Tables 3 and 4). The three-way interaction between condition × nightshifts × circadian type was also not significant (*p =* .143; See Table 3). The main effects of circadian type (*p =* .010) and nightshifts (*p =* < .001) were significant, such that working memory was better in flexible types (58.24 ± 4.95) than rigid types (38.91 ± 4.56) and improved across experimental nights from E1 (43.62 ± 3.6) to E5 (53.67 ± 3.6). The main effects of condition were not significant (*p =* .489).

### Cognitive Performance: Languid-Vigorous

#### Sustained Attention: Psychomotor Vigilance Task

##### Mean Reciprocal Reaction Time (RRT)

The two-way interaction between nightshifts × circadian type was not significant (*p =* .196; See Tables 5 and 6). The main effect of circadian type was not significant (*p =* .197). The main effect of nightshifts was significant (*p* = 0.006), such that the response time was significantly slower on E1 (4.05 ± 0.19) compared to E5 (4.30 ± 0.16; *p =* .002) and on E3 (4.12 ± 0.19) compared to E5 (*p =* .023).

**Table 5.** Results from the mixed effects analysis of variance (ANOVA) showing the main effects of Nightshift (E1–E5), circadian type (LV), and the interaction effects of circadian type by nightshifts.

	Nightshifts		Circadian Type		Circadian Type*Nightshifts	
Languid-Vigorous	F_(df)_	*p*	F_(df)_	*p*	F_(df)_	*p*
**PVT**						
Mean RRT	3.69_(4, 272.1)_	**0.006**	1.86_(1, 12.4)_	0.197	1.52_(4, 270.8)_	0.196
Lapses	0.71_(4, 280.3)_	0.587	0.18_(1, 14.1)_	0.678	0.58_(4, 275.4)_	0.674
**DSST**						
Correct Responses	3.00_(4, 280.3)_	**0.019**	0.02_(1, 13.7)_	0.876	0.70_(4, 276.5)_	0.590
**Stroop**						
Percentage Correct	1.15_(4, 255.2)_	0.330	0.26_(1, 11.9)_	0.615	1.04_(4, 251.8)_	0.385
Reaction Time	9.22_(4, 251.8)_	**<0.001**	0.08_(1, 11.5)_	0.777	6.71_(4, 250.2)_	**<0.001**

*Note*. PVT = Psychomotor Vigilance Task; RRT = reciprocal reaction time; DSST = Digit Symbol Substitution Task. Statistical significance set at *p* = 0.05.

**Table 6.** Means and 95% confidence intervals for all cognitive performance outcomes for languid BREAK and vigorous BREAK for each nightshifts.

Nightshifts		
	Languid	Vigorous
**PVT (Mean RRT)**		
E1	3.72(3.11–4.33)	4.39(3.78–5.00)
E2	3.87(3.26–4.48)	4.39(3.78–4.99)
E3	3.85(3.25–4.46)	4.38(3.77–4.99)
E4	3.89(3.99–4.49)	4.46(3.85–5.07)
E5	4.10(3.28–4.71)	4.51(3.90–5.12)
**PVT (Lapse count)**		
E1	3.74(0.89–6.59)	1.96(−0.90–4.82)
E2	2.19(−0.67–5.06)	2.51(−0.19–5.22)
E3	3.62(0.90–6.33)	2.37(−0.37–5.13)
E4	2.45(−0.32–5.23)	2.00(−0.85–4.84)
E5	1.80(−1.14–4.75)	1.36(−1.56–4.29)
**Stroop (Percentage correct)**		
E1	97.90(96.6–99.3)	98.36(97.1–99.6)
E2	97.60(96.3–99.0)	98.32(97.1–99.5)
E3	97.36(95.9–98.6)	98.14(96.9–99.4)
E4	97.62(96.3–99.0)	98.03(96.7–99.2)
E5	97.66(96.7–99.0)	96.82(96.3–98.8)
**Stroop (Mean response time)**		
E1	**0.93(0.76–1.10)**	0.86(0.70–1.02)
E2	**0.83(0.66–1.01)**	0.85(0.69–1.01)
E3	**0.78(0.61–0.95)**	0.87(0.71–1.01)
E4	**0.80(0.63–0.97)**	0.86(0.70–1.02)
E5	**0.74(0.57–0.91)**	0.80(0.64–0.96)
**DSST (Number of correct responses)**		
E1	42.91(27.7–58.1)	45.27(30.0–60.4)
E2	44.82(29.6–60.0)	45.52(30.7–60.3)
E3	45.20(30.4–68.4)	39.49(24.5–54.3)
E4	53.68(38.7–68.6)	49.36(34.1–64.5)
E5	52.01(36.6–67.4)	51.47(36.3–66.6)

*Note*. PVT = Psychomotor Vigilance Task; RRT = reciprocal reaction time; DSST = Digit Symbol Substitution Task; E1-E5 = Nightshifts 1–5. Numbers in bold indicate a level of statistical difference: *p* ≤ 0.05.

##### Lapse Count

The 2-way interaction between nightshifts × circadian type was not significant (*p =* .674; See Table 5; See Table 6) The main effects of circadian type (*p =* .678) and nightshifts (*p* = 0.587) were not significant.

#### Response Inhibition: Stroop

##### Percentage Correct

The 2-way interaction between nightshifts × circadian type was not significant (*p =* .382; See Tables 5 and 6). The main effects of circadian type (*p =* .615) and nightshifts (*p* = 0.330) were not significant.

##### Mean Response Time

**Figure 5.**
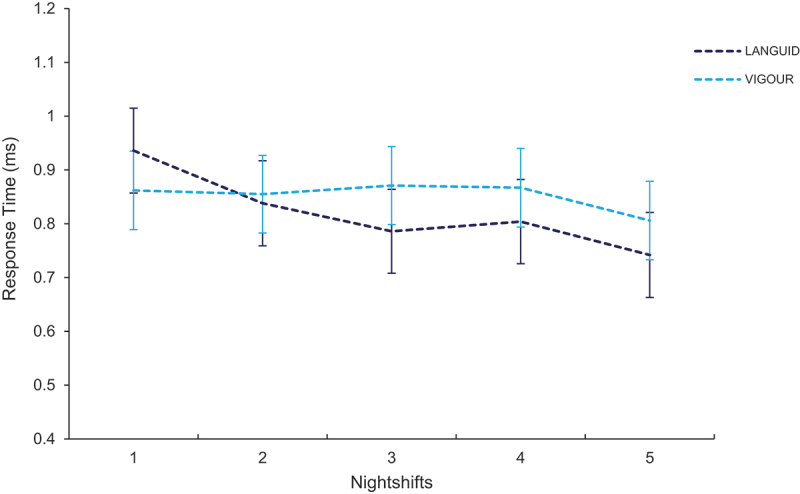
Response time (ms) on the Stroop task across five consecutive nights for languid and vigour types. Error bars presented as standard error.

The 2-way interaction between nightshifts × circadian type was significant (*p =* < .001; See Figure 5, and Tables 5 and 6). Response time was significantly improved in languid types from E1 to E2 (*p* = 0.002), E3 (*p* = <.001), E4 (*p* = <.001) and E5 (*p* = <.001). Conversely, for the vigorous types there was no significant change over the five nights. The main effects of circadian type (*p =* .777) were not significant. The main effects of nightshifts were significant (*p* = <.001), such that the response time was significantly faster from: E1 (0.90 ± 0.05) to E2 (0.85 ± 0.05; *p* = 0.17), E1 to E3 (0.83 ± 0.05; *p* = <.001), E1 to E5 (0.77 ± 0.05; *p* = <.001), E2 (0.84 ± 0.05) to E5 (*p* = 0.013), and E4 (0.83 ± 0.05) to E5 (*p* = 0.014).

#### Working Memory: Digit Symbol Substitution Task

##### Number of Correct

The 2-way interaction between condition × nightshifts × circadian type was not significant (*p =* .590; See Tables 5 and 6). The main effect of circadian type was not significant (*p =* .876). The main effect of nightshifts was significant (*p =* .019), such that the working memory was worse on E3 (42.33 ± 4.89) than E4 (51.54 ± 4.97; *p* = 0.025).

## Discussion

Our study was the first to investigate the relationship between circadian type and breaking up sitting on cognitive performance over five consecutive simulated nightshifts. A flexible circadian type was not associated with an improvement in selected markers of cognitive performance over five nights, with the exception of accurate response inhibition. However, when combined with the breaking up sitting intervention, flexibility was associated with significantly better sustained attention across all five nightshifts relative to rigid types in the breaking up sitting condition, as well as rigid and flexible types in the sedentary condition. No such differences existed for response inhibition, response time or working memory. Our study did not find significant differences between languid and vigorous types with respect to performance trends over five nightshifts for either sustained attention, response inhibition or working memory. However, response times in the response inhibition task improved across consecutive nightshifts among languid types, with no change among vigorous types.

Our major finding for research question 1b. was that combining a flexible circadian type with the breaking up sitting intervention significantly improved sustained attention compared to the performance of flexible types in the sedentary condition, and rigid types in either condition. Performance was worse on the first night for all groups except rigid sedentary types, but did not improve past the second night. The difference between the flexible breaking up sitting condition and all other groups was stable and maintained across all five experimental nightshifts. This lack of change across nights in flexible and rigid types in either activity condition might have been driven by the bright lighting within our laboratory (>300 lux), acting as both an alerting factor and as a promotor of circadian adaption (Sunde et al. 2020), and/or the adequate sleep experienced by flexible (7.9 h) and rigid (7.2 h) types (Hirshkowitz et al. 2015). Though the differences between groups were small, the observed improvement may have implications for tasks overnight which require long bouts of focus. Breaking up sitting could support sustained attention for flexible types, however, alternative countermeasures might be necessary to support sustained attention in rigid types. No effects were observed for the other markers of cognitive performance. Theoretically, it might be expected that flexible types outperform rigid types, given rigidity is associated with less tolerance of altered sleep-wake routines (Di Milia et al. 2005; Saksvik et al. 2011), and flexibility is associated with greater resilience and coping under the same conditions (Di Milia and Folkard 2021; Di Milia et al. 2004, 2005; Saksvik et al. 2011; Wu et al. 2022). This is especially pertinent given that the significant result was related to a task that is sensitive to circadian misalignment (Chellappa et al. 2019). However, it is also possible that we did not find significant effects for the Stroop and Digit Symbol Substitution tasks as short and complex tasks tend to elicit compensatory behaviours that mask fatigue and sleepiness (Sadeghniiat-Haghighi and Yazdi 2015; Yang et al. 2009). Moreover, the intensity of the physical activity in the intervention may not have been sufficiently high to result in detectable changes in cognitive tasks as higher-intensity physical activity elicits longer post-activity benefit (Basso and Suzuki 2017; Basso et al. 2015; Chang et al. 2012; Dishman et al. 2006; Gligoroska and Manchevska 2012). Future research should explore whether higher intensity physical activity during night work is feasible and more effective for both rigid and flexible types.

The mechanism behind the improvement in flexible types following breaking up sitting on a task sensitive to circadian disruption (Chellappa et al. 2019) is worth further exploration. One suggested mechanism is the influence that physical activity has on the timing of behavioural and hormonal circadian rhythms (Buxton et al. 2003; Klerman et al. 1998; Kuwamizu et al. 2022; Shen et al. 2023; Youngstedt et al. 2019), as well as on molecular circadian clocks in peripheral tissues (Gabriel and Zierath 2019; Hansen et al. 2016; Martin and Esser 2022; Zambon et al. 2003). Flexible types may be coping better with circadian disruption and rhythm instability, as well as receiving a greater benefit from the physical activity, with the combination resulting in a phase shifting effect. While we did not collect physiological measures (e.g. hormonal melatonin, core body temperature) in the current study, future research could incorporate these or other non-invasive measures (e.g. wearables, heart rate variability) to track circadian and physiological responses. These additional measures would enhance interpretation of the current findings and allow for a better understanding of individual differences (e.g. circadian type) in response to breaking up sitting.

Our results for research question 1a. showed that, aside from accurate response inhibition, trends in cognitive performance did not differ between flexible and rigid circadian types over five nights independent of breaking up sitting. However, a rigid circadian type was associated with decreased accuracy. Whether this 1.4% decrease from night one to five has meaningful real-world implications must be considered. The lack of sleep restriction overall may not be reflective of flexible and rigid shift workers experiencing poor sleep outcomes (Chellappa et al. 2019; Kecklund and Axelsson 2016; Niu et al. 2011).

Our findings for research question 2a. show that, aside from response time on the Stroop task, neither languidity nor vigour was associated with discernible performance differences over five consecutive nightshifts. Data revealed response time on the Stroop task improved across days for languid types but not vigorous types. This result was surprising given that vigorous types report less daily sleep need (Di Milia and Folkard 2021; Di Milia et al. 2004, 2005) and that languid types are reported to be less resilient to the effects of sleep loss (Di Milia and Folkard 2021; Di Milia et al. 2005, 2013; Jafari Roodbandi et al. 2015; Saksvik et al. 2011). It is possible that languid types received greater benefit from the breaking up sitting intervention such that performance was equivalent to vigorous types. However, without the sedentary control group, we cannot confirm this supposition. It is more likely that because languid types did not experience sleep restriction (7.7 h), relative to vigorous types (6.8 h), performance was similar or superior in the former group. We could not answer research question 2b as participants were not screened based on circadian type resulting in low sample numbers in the SIT condition for the languid subgroup. As such, differences in response to breaking up sitting between languid and vigorous types could not be evaluated. However, our results provide potentially important information regarding sleep characteristics and sleep need between languid and vigour categorisations (Di Milia et al. 2005).

Our study was the first to explore circadian type and breaking up sitting with light-intensity physical activity, and a study strength lies in the randomised experimental design and highly controlled protocol. However, limitations exist within our study. As the present study was part of a broader project, this secondary analysis did not include allocation of participants to conditions based on circadian type. Though the principal study was powered for the primary cardiometabolic outcomes, the lack of allocation based on circadian type in the present study resulted in unequal groups and small sample sizes. While we found effects, no adjustments for multiple comparisons were made to analyses due to the conservative nature of these corrections (Nakagawa 2004). As such, there is a chance of Type I errors (Armstrong 2014), and our results should be interpreted with caution. While our study examined how breaking up sitting impacted performance between circadian types over consecutive nightshifts, we did not examine the interventions influence on performance declines that typically occur within a night shift. Future research is needed to explore within-shift performance to better understand the efficacy of breaking up sitting and the role of circadian type during individual nightshifts. Although body mass index and waist-to-hip ratio were used as inclusion criteria for the principal study, no height criteria existed which resulted in height differences between languid and vigorous types. Total number of steps was significantly different as a result of height differences, and the consequent stride length differences are a limitation. Finally, the short-term laboratory nature of this design means that the long-term influence of circadian type and physical activity on cognitive performance in real shift workers cannot be interpreted.

## Conclusion

Our study is the first randomised controlled trial to explore the impact of circadian type, and circadian type and breaking up sitting, on cognitive performance during simulated nightshifts. Breaking up sitting maintained sustained attention in flexible types as compared to rigid types. Overall, our results indicate that circadian type classifications should be accounted for in breaking up sitting interventions.

## Data Availability

Data will be made available on request.
